# The effect of JuanBiQiangGu granules in combination with methotrexate on joint inflammation in rheumatoid arthritis: a randomized controlled trial

**DOI:** 10.3389/fphar.2023.1132602

**Published:** 2023-04-19

**Authors:** Lei Ran, Bo Xu, Hai-Hui Han, Jian-Ye Wang, Xin-Yu A, Bo-Ran Cao, Xiao-Hui Meng, Cheng-Bo Zhang, Peng-Fei Xin, Guo-Wei Qiu, Zheng Xiang, Shao-Qiang Pei, Chen-Xin Gao, Jun Shen, Sheng Zhong, Xi-Rui Xu, Yan-Qin Bian, Jun Xie, Qi Shi, Song-Tao Sun, Lian-Bo Xiao

**Affiliations:** ^1^ Guanghua Clinical Medical College, Shanghai University of Traditional Chinese Medicine, Shanghai, China; ^2^ Department of Orthopedic Surgery, Shanghai Guanghua Hospital of Integrated Traditional Chinese and Western Medicine, Shanghai, China; ^3^ Institute of Arthritis Research in Integrative Medicine, Shanghai Academy of Traditional Chinese Medicine, Shanghai, China

**Keywords:** JuanBiQiangGu granules, methotrexate, rheumatoid arthritis, traditional Chinese medicine, joint inflammation

## Abstract

**Background:** Rheumatoid arthritis (RA) joint inflammation severely affects joint function and quality of life in patients and leads to joint deformities and limb disability. The non-steroidal anti-inflammatory drugs used in the treatment of RA do not fully control the progression of joint inflammation and bone destruction and have notable adverse reactions. Traditional Chinese medicine formula JuanBiQiangGu Granules (JBQG) are commonly used for the treatment of RA inflammation and delay of bone destruction, but has not been evaluated through high-quality clinical studies. There is a pressing need for well-designed, randomized, parallel, controlled clinical studies to evaluate the exact effect of JBQG on RA joint inflammation and improvement of patient quality of life.

**Methods:** This is a randomized, parallel, controlled clinical study in which 144 patients with rheumatoid arthritis who met the inclusion criteria were randomly assigned to 2 groups in a 1:1 ratio. The JBQG group received methotrexate 7.5 mg qw and JBQG granules 8 mg tid, while the MTX group received methotrexate 7.5 mg qw. The endpoint was 12 weeks after treatment. Relevant indices at baseline, 4 weeks, 8 weeks, and 12 weeks after treatment were observed and recorded, and DAS28-ESR, HAQ-DI, and Sharp scores were recorded for each patient. Blood samples were collected to test for CRP, ESR, TNF-α, IL-1β, IL-6, IL-17, and INF-γ, and adverse reactions and liver and kidney function (AST, ALT, Cr, BUN) were recorded for safety assessment. After 12 weeks of treatment, the effect of JBQG granules on disease activity, improvement in bone damage, and patient quality of life scores and safety in RA patients were evaluated.

**Results:** A total of 144 subjects completed treatment (71 in the JBQG group and 73 in the MTX group) and were included in the analysis. At baseline, there were no significant differences between the groups in terms of the observed indicators (*p* > 0.05). After treatment, 76.06% of patients in the JBQG group had DAS28-ESR levels below or equal to Low, including 45.07% in Remission and 5.63% in High, compared to 53.1% in the MTX group below or equal to Low, 12.33% in Remission, and 17.81% in High. CRP was significantly reduced (8.54 ± 5.87 vs. 11.86 ± 7.92, *p* < 0.05, *p* = 0.005), ESR was significantly reduced (15.1 ± 6.11 vs. 21.96 ± 9.19, *p* < 0.0001), TNF-α was significantly reduced (1.44 ± 0.83 vs. 1.85 ± 1.07, *p* < 0.05, *p* = 0.011), IL-17 was significantly reduced (0.53 ± 0.33 vs. 0.71 ± 0.38, *p* < 0.05, *p* = 0.004), and INF-γ was significantly reduced (3.2 ± 1.51 vs. 3.89 ± 1.77, *p* < 0.05, *p* = 0.014). The median (IQR) OPG in the JBQG group was 2.54 (2.21–3.01), significantly higher than in the MTX group 2.06 (1.81–2.32), *p* < 0.0001), and the median (IQR) *β*-CTX in the JBQG group was 0.4 (0.32–0.43), significantly lower than in the MTX group 0.55 (0.47–0.67), *p* < 0.0001). The median (IQR) VSA scores were 2 (1–3), a decrease from 3 (2–4) in the MTX group (*p* < 0.0001). The median (IQR) Sharp scores were 1 (1–2), a decrease from 2 (1–2) in the MTX group, but the difference was not statistically significant (*p* > 0.05, *p* = 0.28). The median (IQR) HAQ-DI scores were 11 (8–16), significantly lower than in the MTX group 26 (16–30) (*p* < 0.0001). The median (IQR) AST in the JBQG group was 16 (12–20), with a significant difference compared to the MTX group 19 (13–25) (*p* < 0.01, *p* = 0.004); the median (IQR) ALT in the JBQG group was 14 (10–18), with a significant difference compared to the MTX group 16 (11–22.5) (*p* < 0.05, *p* = 0.015). There were no statistically significant differences in Cr or BUN (*p* > 0.05).

**Conclusion:** JuanBiQiangGu Granules can be used to treat patients with rheumatoid arthritis, alleviate joint inflammation, reduce the incidence of adverse reactions to methotrexate, and has good safety.

**Clinical Trial Registration:** http://www.chinadrugtrials.org.cn/index.html; identifier: ChiCTR2100046373.

## Introduction

Rheumatoid arthritis (RA) is a chronic autoimmune disease that often presents with asymptomatic joint inflammation in its early stages, which progresses and exacerbates with repeated joint inflammation and cartilage damage until irreversible joint damage occurs, leading to complications in multiple organs throughout the body, resulting in severe physiological and psychological dysfunction (Chaurasia et al., 2020; Giannini et al., 2020). The incidence of RA varies significantly among different races and ethnicities, with a higher risk among whites and a lower risk among Asians and African Americans (Conforti et al., 2021). In China in 2018, the incidence of RA was approximately 0.42%, with an estimated 6 million potential patients (Tian X et al., 2021).

The exact mechanisms of RA are not fully understood, but research suggests that the complex interplay between genetics, environmental factors, and immune system responses may lead to the development of RA (Molendijk et al., 2018; Mueller et al., 2021). A key feature of the mechanism of RA is autoimmune response, in which the immune system produces antibodies against self, which attack normal body tissue, leading to tissue damage and joint inflammation (Scherer et al., 2020). This immune response may be due to genetic factors, environmental factors (such as infection), or a combination of both (Liu E and Perl, 2019). RA is also related to the response of cytokines and inflammatory cytokines. Cytokines are proteins that can promote cell growth, division, and function. In RA, the levels of cytokines such as interleukin-1 (IL-1), tumor necrosis factor-alpha (TNF-α), and interferon-gamma (IFN-γ) increase, which can promote cell activation and inflammation. RA is also associated with autoantibodies (Lin et al., 2020; Kondo et al., 2021). In RA, anti-cyclic citrullinated peptide antibodies (ACPA) are commonly produced. ACPA can bind to cyclic citrullinated proteins, leading to joint damage and inflammation (Van Delft and Huizinga, 2020; Li et al., 2021).

Currently in China, the first-line drugs used to improve the condition are disease-modifying antirheumatic drugs (DMARDs) (Jiang, 2020; Tian X P et al., 2021). Although many biologics have emerged in recent years with good clinical efficacy targeting different targets, biologics still have the risk of inducing tumors, hepatitis, tuberculosis, and other diseases and cannot completely replace other treatment methods, and are often used in combination with other treatment methods (Smolen et al., 2020; Tong, 2022). RA requires lifelong or long-term drug use to control it, and many RA patients in China cannot afford long-term drug costs. At the same time, the specificity and relatively high cost of biologics limit their use. Chinese medicine treatment for RA has a long history in China, but the use of Chinese medicine alone still has the possibility of RA joint inflammation recurrence, and it is now often used in combination with traditional DMARDs (Xu et al., 2018; Zuo et al., 2018; Zhang Y et al., 2021).

JuanBiQiangGu Granules is a Chinese medicine formula that has been used for decades in China, consisting of *Psoralea corylifolia Linn*, *Epimedium brevicornu Maxim*, *Davallia mariesii T. Moore ex Baker*, *Sinomenii Caulis*, and *Angelicae Sinensis Radix*. We have observed in clinical practice that JBQG can improve patients’ joint inflammation and reduce inflammation activity and the adverse reactions of DMARDs. *In vitro* experiments have shown that the JBQG drug serum can inhibit the expression of cytokines (IL-6, IL-8, IL-22, IL-23), chemokine proteins and mRNA expression (MMP-1, MMP-2, MMP-3, MMP-9, MMP-13) in rheumatoid arthritis synovial fibroblasts (RA-FLS). *In vivo* experiments have verified through Micro-CT that JBQGF reduces the loss of bone mass around the joints of CIA rats (Gao et al., 2012; Gao et al., 2013; Xie et al., 2020).

However, JBQG has lacked high-quality evidence-based evidence to support its effectiveness and treatment effect has been difficult to evaluate. Therefore, we conducted a randomized controlled trial (RCT) to evaluate the effectiveness and safety of JBQG and to assess whether JBQG can reduce the side effects of DMARDs such as methotrexate.

## Methods

### Trial design

We designed this randomized, parallel, controlled trial and conducted it under the principles of Good Clinical Practice guidelines and the Helsinki Declaration. The trial protocol was registered with the Chinese Clinical Trial Registry (www.chinadrugtrials.org.cn, Registration Number: ChiCTR2100046373) and was reviewed and approved by the Ethics Committee of Shanghai Guanghua Hospital of Integrated Traditional and Western Medicine. All patients provided written informed consent and agreed to sign it. This study followed the CONSORT guidelines for conducting the trial.

### Participants

**FIGURE 1:**
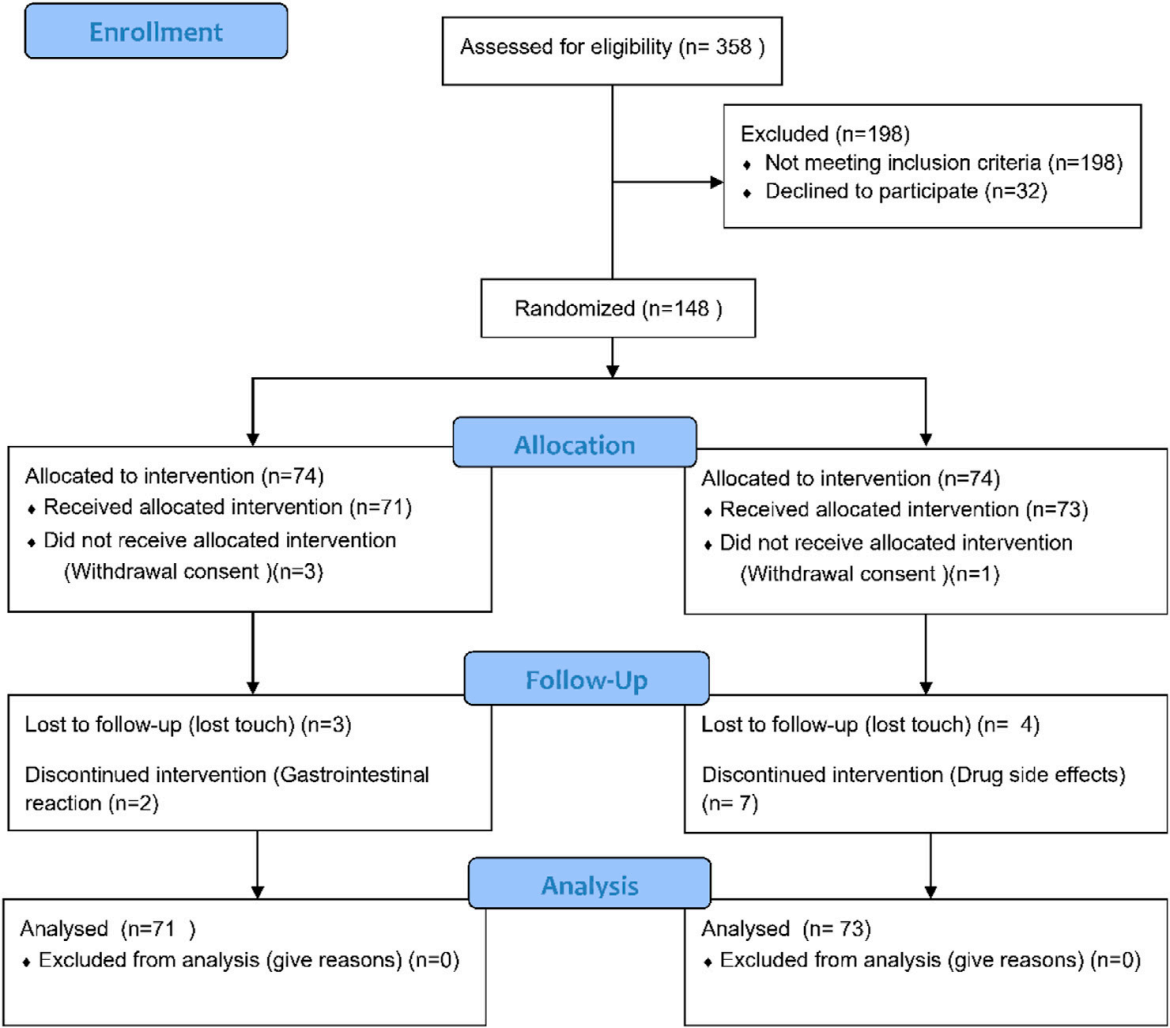
CONSORT flow diagram.

The study participants were adult Chinese citizens aged 20–80 years diagnosed with RA according to the 2009 ACR/EULAR diagnostic criteria and laboratory examination between March 2021 and September 2022 at Shanghai Guanghua Hospital of Integrated Traditional and Western Medicine, with a BMI between 18.0 and 30.0 kg/m2 and no gender limitation. Baseline vital signs, medical history, and DAS-ESR scores < 3.2 were collected, and the physician evaluated whether the patient was suitable for the study. Patients with the following conditions were excluded and could not participate in the study: 1) abnormal liver and kidney function; 2) pregnant or lactating women or patients with mental illness; 3) patients with serious diseases such as heart, liver, brain, kidney, and hematopoietic system; 4) patients with overlap of other rheumatic diseases such as systemic lupus erythematosus, Sjögren’s syndrome, and severe knee osteoarthritis; 5) late stage patients and patients with severe joint deformity; 6) those who received other research drugs in the previous 3 months; 7) those who cannot or do not want to provide informed consent or cannot comply with the study requirements; 8) all subjects deemed inappropriate to participate in the study by the researchers. Figure 1 shows the entire screening process.

### Intervention

The JBQG granules used in this study are in-hospital formulations from the Shanghai Guanghua Hospital of Integrated Chinese and Western Medicine, and are produced by Shanghai Leiyushang Pharmaceutical Co., Ltd. They consist of 15 g of *P. corylifolia Linn*, 15 g of *D. mariesii T. Moore ex Baker*, 10 g of *E. brevicornu Maxim*, 7.5 g of *Sinomenii Caulis*, and 6 g of *Angelicae Sinensis Radix*. According to traditional Chinese medicine theory, they have the functions of nourishing the kidneys and strengthening the bones, activating blood circulation and resolving stasis, and reducing swelling and relieving pain. The JBQG group received methotrexate 7.5 mg qw and 8 g of JBQG granules tid. The MTX group received methotrexate 7.5 mg qw. The observation period was 12 weeks. Baseline indices were collected at enrollment, and then laboratory indices (CRP, ESR, TNF-α, IL-1β, IL-6, IL-17, INF-γ) and various scores (DAS-28, HAQ-DI, Sharp score) were collected every 4 weeks, and the occurrence of adverse reactions and liver and kidney function indices were recorded for safety evaluation. All assessments and radiological examinations were conducted by the Department of Laboratory Medicine and Department of Radiology at Shanghai Guanghua Hospital of Integrated Chinese and Western Medicine. A dedicated person was responsible for patient information collection and follow-up investigation.

### Randomization

An independent person used the random number generator in SPSS 26.0 with a fixed value of 20,210,301 to generate random numbers. After the cases were ranked, two groups were generated. The independent person placed the generated grouping and numbering in opaque envelopes. The document management personnel and the drug distribution personnel were not informed of the specific grouping situation before the end of the study. The data collection and preservation were completed by independent personnel without knowing the experimental grouping situation.

### Sample size calculation

This study is a randomized controlled trial with the primary endpoint being the remission rate of DAS28-ESR. Based on previous clinical observations and literature reports, the remission rate in the traditional Chinese medicine group is approximately 76.6%, whereas the remission rate in the MTX group is approximately 50%. With a two-sided alpha of 0.05 and a power of 90%, the sample size for the traditional Chinese medicine group is N1 = 60 cases and the sample size for the control group is N2 = 60 cases. Taking into account the possibility of dropouts and refusals, a total of at least 72 cases are needed in each group, resulting in a minimum of 144 cases included in the study.

### Quality control

**FIGURE 2:**
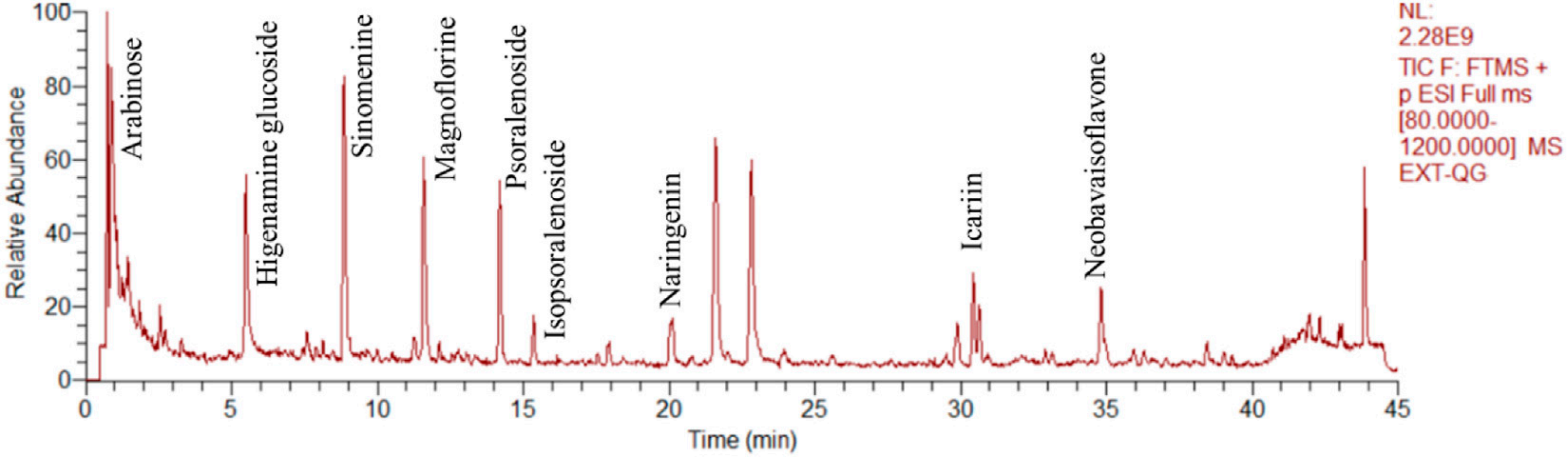
Total ion chromatograms of JBQG in UHPLC-Q-Orbitrap HRMS negative mode and positive mode were obtained.

The herbal ingredients, *P. corylifolia Linn, E. brevicornu, Davallia trichomanoides Blume, Caulis Sinomenii,* and *Angelica sinensis*, in the JuanBiQiangGu granules are all from Shanghai Tongjitang Pharmaceutical Co., Ltd., with batch numbers 220701, 220,701, 220,501, and 220,301, and should be stored away from light. The JuanBiQiangGu granule extract powder is produced by Shanghai Traditional Chinese Medicine Pharmaceutical Co., Ltd. The production process involves taking 15 kg of *P. corylifolia Linn*, 15 kg of *E. brevicornu Maxim*, 7.5 kg of *Sinomenii Caulis*, 6 kg of *Angelicae Sinensis Radix*, and 10 kg of *D. mariesii T. Moore ex Baker*, and placing them in an extraction tank. The mixture is boiled three times, with each round using 8 times the amount of water, boiling for 1 h, and filtering through a 200-mesh sieve. The filtered solution is then vacuum concentrated to a relative density of 1.25–1.30 (60°C) paste, which is then filtered and set aside. The paste is then taken and placed in a vacuum dryer, controlling the thickness of the material to be no more than 0.8 cm, and drying it at a temperature of 60°C–80°C and a vacuum degree of −0.06 Mpa ∼ -0.10 Mpa until the water content of the dried extract is no more than 5%. The dried extract is then powdered and filtered through a 100-mesh sieve and set aside. The extract powder is vacuum packed and stored away from light. Depending on the concentration of the final extract powder, a solution of 2.2 g/mL or 4.4 g/mL is prepared with pure water for immediate use. The quality control of Juanbi Qianggu granules follows the testing methods specified in the “Chinese Pharmacopoeia 2020”. The results of the tests show that the total content of psoralen and isopsoralen in *P. corylifolia Linn* is 0.70%, the content of hesperidin in *E. brevicornu* is 0.86%, the content of ferulic acid in *A. sinensis* is 0.072%, the total content of icariin, icaritin, and desmethylicaritin, and epimedin in *D. trichomanoides Blume* is 1.5%, and the content of corynoline in *Caulis Sinomenii* is 1.09%. All of the above-mentioned crude drugs comply with the quality inspection standards. Figure 2 presents the results of the quality analysis.

## Outcomes

### Primary outcome

The primary endpoint of this study is the rate of DAS28-ESR remission at 12 weeks of treatment. DAS28-ESR (Disease Activity Score 28—Erythrocyte Sedimentation Rate) is a tool used to evaluate the disease activity of patients with rheumatoid arthritis (RA). It consists of the DAS28 score (disease activity index) and ESR (erythrocyte sedimentation rate). The DAS28-ESR score is used to assess the severity of RA by measuring symptoms such as joint swelling, pain, functional impairment, and pain scores. ESR is a common blood test that helps doctors evaluate the level of inflammation. The DAS28-ESR score is used to assess the change in treatment effectiveness in RA patients, and can assess the sustainability, inter-individual differences, and reliability of treatment effectiveness in RA patients. Remission is defined as a DAS28-ESR score of less than 2.6; low is less than 3.2; moderate is greater than or equal to 3.2 and less than or equal to 5.1; and high is greater than 5.1. We will determine the stage of RA based on the final DAS28-ESR score and guide patients in adjusting their treatment regimen accordingly.

## Secondary clinical efficacy outcomes

The secondary outcome measures include cytokines, including TNF-α, IL-1β, IL-6, IL-17, and INF-γ, in the blood of RA patients. Cytokines are a class of proteins produced by cells that regulate immune and inflammatory responses. They are present at low levels in the blood of healthy individuals, but can increase in RA patients due to inflammation or tissue damage, such as increases in CRP and ESR levels. CRP is a protein produced by the liver and transported through the bloodstream to the body’s tissues, where it increases in response to inflammation or tissue damage. ESR is the rate at which red blood cells settle in a low concentration of hydrochloric acid solution. Inflammation or tissue damage can cause substances such as leukocyte interleukins and transforming growth factors to be produced, resulting in the clumping of red blood cells and an increase in the rate of red blood cell sedimentation. Therefore, measuring the values of CRP and ESR in RA patients can help doctors assess disease activity and progression. The Health Assessment Questionnaire Disability Index (HAQ-DI) is a tool used to assess functional impairments in RA patients. It consists of 20 questions related to daily activities such as bathing, dressing, and walking. Patients evaluate the assistive tools or help needed to perform each activity and score it. The scores for all questions are then added up to give the HAQ-DI score. The lower the HAQ-DI score, the smaller the functional impairments of the patient. This tool helps doctors assess functional impairments in RA patients as well as monitor the progression of the disease and provides a basis for clinical decision-making.

The Sharp score is a tool used to assess the severity of bone damage in patients with rheumatoid arthritis (RA) by evaluating the results of imaging exams and the extent of joint damage. The Sharp score ranges from 0 to 448, with higher scores indicating a higher degree of cartilage damage (Han et al., 2018). Safety indicators mainly involve tracking abnormal reactions and liver and kidney function markers (AST, ALT, Cr, BUN) between the two groups. If there are any abnormal circumstances, patients will be promptly withdrawn from the study and other treatment plans, such as biologic therapy, other anti-rheumatic drugs, and glucocorticoids, will be intervened based on the different disease presentations.

### Statistical analysis

Statistical analysis was conducted using SPSS Statistics 26 (IBM), and graphs were plotted using GraphPad Prism 9.5.0 (www.graphpad.com). Data were organized using Microsoft Excel. The Kolmogorov-Smirnov test (K-S test) is first applied to all quantitative data to determine whether they follow a normal distribution. Descriptive data that were normally distributed were expressed as X̅ ± S and compared between groups using a *t*-test, or a t’-test if the variance was not equal. Data that were not normally distributed were described as the median and interquartile range, and compared between groups using a Mann-Whitney U test. A *p*-value < 0.05 was considered statistically significant.

The intention-to-treat (ITT) principle was employed to analyze all randomized cases. The full analysis set (FAS) was used for all outcome measures, excluding cases that withdrew from treatment after randomization. Cases should include complete baseline data, and those who significantly violated or seriously violated the clinical trial protocol were excluded. All cases participating in drug treatment were included in the analysis. If the proportion of missing, dropped, or withdrawn case-related data is less than 10% of the total enrolled cases, missing values are not imputed. If this value is greater than 10%, multiple imputation methods are used to impute missing data values.

## Results

From March 2021 to September 2022, a total of 358 RA patients were screened, of which 198 were excluded, 32 did not agree to participate in the study, and 148 were finally enrolled and randomly divided into the JBQG group (n = 74) and the MTX group (n = 74). Figure 1 shows the process of the 144 patients who completed the study and were finally analyzed. A total of 95.1% (n = 137) of patients completed 12 weeks of treatment, 95.8% (n = 68) received JBQG 8 g tid + MTX 7.5 mg qw treatment, and 94.5% (n = 69) received MTX 7.5 mg qw treatment. In addition, we conducted regular follow-up assessments. After 36 weeks of treatment, 76.1% (n = 54) of the JBQG group and 60.3% (n = 44) of the MTX group received follow-up. After 60 weeks of treatment, 49.3% (n = 45) of the JBQG group and 43.8% (n = 32) of the MTX group received follow-up. Although follow-up data are not included in the analysis of this study, we non-etheless conducted tracking and recording to facilitate retrospective analysis for other research projects.

In the final analysis of 144 patients, the two groups of patients were similar in terms of demographic and clinical characteristics (Table 1). The median age (IQR) of the JBQG group was 60.5 (51.25–66) years and the median age (IQR) of the MTX group was 62 (53–68.5) years; 116 patients (80.6%) with RA were female. There were no statistical differences between the two groups in terms of BMI, past medical history, baseline cytokines, and quality of life scores (*p* > 0.05), and the baseline measurements were similar between the two groups (Table 1).

**TABLE 1:** Baseline characteristics of two patient groups.

Characteristic	JBQG group	MTX group	*p*-Value
	(n = 71)	(n = 73)	
Age [years; median (IQR)]	60 (51–66)	63 (55–69)	0.254^a^
Gender [n (%)]			0.615^b^
Male	15 (21.12)	13 (17.80)	
Female	56 (78.87)	60 (81.19)	
BMI [kg/m^2^;mean (SD)]	23.25 (3.75)	23.31 (3.23)	0.405^c^
Past medical history [n (%)]			0.723^b^
Hypertension	42 (60.56)	39 (52.05)	
Diabetes	21 (29.58)	23 (31.51)	
Cardiac disease	8 (11.27)	11 (15.07)	
Other autoimmune diseases	00)	00)	
Average disease duration [years; mean (range)]	7.2 (0.4–14.5)	8 (0.3–15.5)	0.124^a^
The history of biologics use^*^ [n (%)]	24 (33.80)	26 (35.61)	0.819 ^b^
The history of glucocorticoid use^*^ [n (%)]	45 (63.38)	42 (57.53)	0.387 ^b^
Cytokines [mg/L; mean (SD)]			
TNF-α	2.47 (1.43)	2.54 (1.47)	0.765^c^
IL-1β	5.63 (1.85)	5.82 (1.69)	0.522^c^
IL-6	2.54 (0.81)	2.66 (0.83)	0.382^c^
IL-17	0.91 (0.57)	0.97 (0.52)	0.552^c^
INF-γ	4.58 (2.16)	4.89 (2.25)	0.412^c^
OPG [U/L; median (IQR)]	1.91 (1.66–2.26)	1.98 (1.74–2.22)	0.329^a^
*β*-CTX [ng/ml; median (IQR)]	0.48 (0.39–0.55)	0.46 (0.39–0.54)	0.895^a^
CRP [mg/L; mean (SD)]	9.89 (5.49)	10.75 (6.17)	0.375^c^
ESR [mm/H; mean (SD)]	23.65 (10.37)	22.58 (11.20)	0.552^c^
DAS28-ESR [scores; median (IQR)]	2.69 (2.46–2.87)	2.78 (2.51–2.95)	0.308^a^
AST [U/L; median (IQR)]	13 (9–18)	14 (8–17)	0.429^a^
ALT [U/L; median (IQR)]	15 (11–20)	16 (10.25–19)	0.734^a^
Cr [umol/L; median (IQR)]	73 (67–81)	76 (69.5–81)	0.291^a^
BUN [mg/dL; median (IQR)]	13 (11–15)	13 (11–14)	0.881^a^
VAS [scores; median (IQR)]	2 (1–4)	3 (2–4)	0.349^a^
Sharp [scores; mean (SD)]	1 (1–2)	1 (1–2)	0.707^c^
HAQ-DI [scores; mean (SD)]	13 (9–18)	15 (9.5–18)	0.438^c^

a, Mann- Whitney *U* test; b, Person χ^2^;c, *t*-test; *, all participants who have previously received medications such as glucocorticoids and biologics will undergo a 3-month washout period.

Participants and baseline characteristics.

## Primary outcome

**FIGURE 3:**
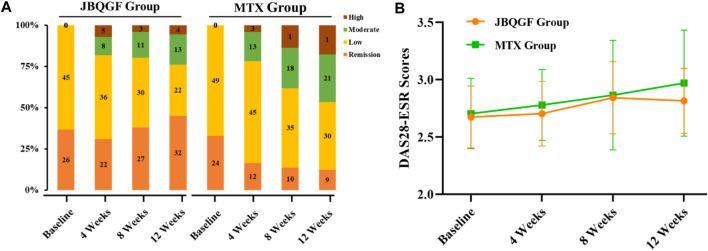
Differences in DAS28-ESR distributions **(A)** and trends in test scores **(B)** between the two groups.

Before treatment, there was no difference in DAS28-ESR between the JBQG group and the MTX group (*p* > 0.05). After 12 weeks of treatment, the median DAS28-ESR score for the JBQG group (IQR) was 2.61 (2.25–3.11), which was significantly lower than that of the MTX group 3.17 (2.95–3.73), with statistical significance (*p* < 0.05, *p* = 0.017). Compared with the MTX group, the JBQG group had a common advantage over different symptoms in terms of remission: 1 for remission; 4.85 (95% CI: 1.93–12.19, *p* = 0.001) for low; 5.74 (95% CI: 2.09–15.81, *p* = 0.006) for moderate; and 11.56 (95% CI: 3.02–44.25, *p* < 0.0001) for high (Table 2; Figure 3).

**TABLE 2:** Main efficacy results (intention-to-treat population).

Outcomes	JBQG group (n = 71)	MTX group (n = 73)		*p*-Value
Primary efficacy outcome				
DAS28-ESR [scores; median (IQR)]	2.61 (2.25–3.11)	3.17 (2.95–3.73)	0.017^a^	
Proportional distribution [n (%)]			OR (95% CI) JBQG group vs. MTX group	*p*-value
<2.6	32 (45.07)	9 (12.33)	1	
<3.2	22 (30.99)	30 (41.10)	4.85 (1.93–12.19)	0.001 ^b^
≥3.2,≤5.1	13 (18.31)	21 (28.77)	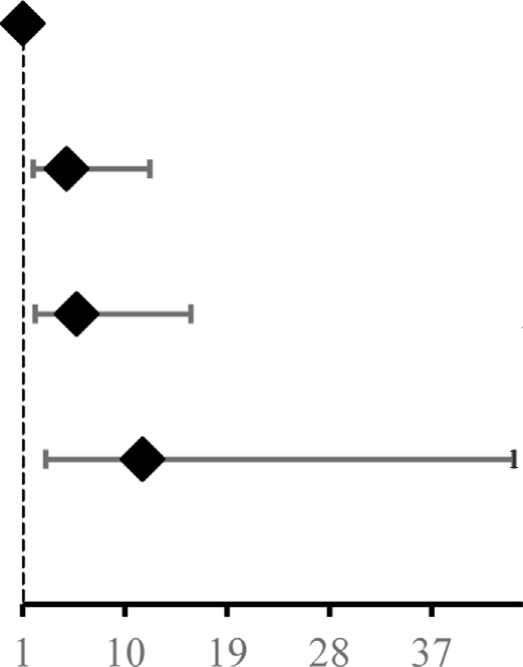 5.74 (2.09–15.81)	0.006 ^b^
>5.1	4 (5.63)	13 (17.81)	11.56 (3.02–44.25)	<0.0001 ^b^
Secondary clinical efficacy outcomes				
Cytokine [mg/L; mean (SD)]			*p*-value	
TNF-α	1.44 (0.83)	1.85 (1.07)	0.011^c^	
IL-1β	4.11 (1.35)	4.24 (1.23)	0.521^c^	
IL-6	2.54 (0.81)	2.67 (0.83)	0.378^c^	
IL-17	0.53 (0.33)	0.71 (0.38)	0.004^c^	
INF-γ	3.20 (1.51)	3.89 (1.77)	0.014^c^	
OPG [U/L; median (IQR)]	2.54 (2.21–3.01)	2.06 (1.81–2.32)	<0.0001^a^	
*β*-CTX [ng/ml; median (IQR)]	0.4 (0.32–0.43)	0.55 (0.47–0.67)	<0.0001^a^	
CRP [mg/L; mean (SD)]	8.54 (5.87)	11.86 (7.92)	0.005^c^	
ESR [mm/H; mean (SD)]	15.1 (6.11)	21.96 (9.19)	<0.0001^c^	
VAS score [scores; median (IQR)]	2 (1–3)	3 (2–4)	<0.0001^a^	
Sharp score [scores; median (IQR)]	1 (1–2)	2 (1–2)	0.28^a^	
HAQ-DI score [scores; median (IQR)]	11 (8–16)	26 (16–30)	<0.0001^a^	
Safety outcome				
AST [U/L; median (IQR)]	16 (12–20)	19 (13–25)	0.004^a^	
ALT [U/L; median (IQR)]	14 (10–18)	16 (11–22.5)	0.015^a^	
Cr [umol/L; median (IQR)]	73 (67–81)	76 (68–84)	0.322^a^	
BUN [mg/dL; median (IQR)]	12 (10–14)	13 (11–14)	0.397^a^	

a, Mann- Whitney *U* test; b, Person χ2; c, *t*-test.

### Secondary outcomes

The JBQG group and MTX group had no differences in the 8 secondary indicators before JBQG treatment (*p* > 0.05). After 12 weeks of treatment, compared to the MTX group, the JBQG group had significantly lower CRP (8.54 ± 5.87 vs. 11.86 ± 7.92, *p* < 0.05, *p* = 0.005), ESR (15.1 ± 6.11 vs. 21.96 ± 9.19, *p* < 0.0001), TNF-α (1.44 ± 0.83 vs. 1.85 ± 1.07, *p* < 0.05, *p* = 0.011), IL-17 (0.53 ± 0.33 vs. 0.71 ± 0.38, *p* < 0.05, *p* = 0.004), and INF-γ (3.2 ± 1.51 vs. 3.89 ± 1.77, *p* < 0.05, *p* = 0.014), but there were no significant differences in IL-1β (4.11 ± 1.35 vs. 4.24 ± 1.23, *p* > 0.05) or IL-6 (2.54 ± 0.81 vs. 2.67 ± 0.83, *p* > 0.05).

The median (IQR) OPG score in the JBQG group was 2.54 (2.21–3.01) which was significantly higher than in the MTX group 2.06 (1.81–2.32) (*p* < 0.0001). The median (IQR) *β*-CTX score in the JBQG group was 0.4 (0.32–0.43) which was significantly lower than in the MTX group 0.55 (0.47–0.67) (*p* < 0.0001). The median (IQR) VSA score in the JBQG group was 2 (1–3) which was lower than in the MTX group 3 (2–4) (*p* < 0.0001). The median (IQR) Sharp score in the JBQG group was 1 (1–2) which was not significantly different from the MTX group 2 (1–2) (*p* > 0.05). The median (IQR) HAQ-DI score in the JBQG group was 11 (8–16) which was significantly lower than in the MTX group 26 (16–30) (*p* < 0.0001).

## Safety outcomes

In the MTX group, 9.6% (n = 7) of patients experienced adverse events, with the most common reason for withdrawing from the study being drug-related side effects such as leukopenia and abnormal liver and kidney function. In the JBQG group, 2.8% (n = 2) of patients experienced adverse events, all of which were gastrointestinal reactions such as vomiting and nausea. After 12 weeks of treatment, the median (IQR) AST value in the JBQG group was 16 (12–20), which was significantly different from the MTX group of 19 (13–25) (*p* < 0.01, *p* = 0.004). The median (IQR) ALT value in the JBQG group was 14 (10–18), which was significantly different from the MTX group 16 (11–22.5) (*p* < 0.05, *p* = 0.015). There was no statistically significant difference in Cr, and BUN values (*p* > 0.05).

## Discussion

Before the study began, we compared the basic information, such as age, gender, BMI, and medical history, of patients in the JBQG group and the MTX group and found that they were in a comparable state. The ratio of females to males in both groups was roughly between 1: 2.1–2.7, which was similar to the ratio of males to females in a large epidemiological survey. At the same time, the influence of other immune-related diseases was eliminated, but a proportion of the patients had previously used corticosteroids and biological preparations. For these patients, we discontinued the relevant drugs for more than 12 weeks. There were no statistically significant differences between the two groups in the HIQ-DI, VAS scores, and Sharp scores (*p* > 0.05). After 12 weeks of treatment, we found that the control of DAS28-ESR in the JBQG group was good, with a median (IQR) of 2.61 (2.25–3.11), which was significantly lower than that in the MTX group 3.17 (2.95–3.73), and the difference was statistically significant (*p* < 0.05, *p* = 0.017). In addition, the proportions of Remission, Low, Moderate, and High were significantly different from those in the MTX group. The proportion of Remission (< 2.6) in the two groups was 45.07% vs. 12.33%, indicating the good control of remission period in the JBQG group and the significant increase in the proportion of remission period patients. In addition, the proportion of High (>5.1) in the two groups was 5.63% vs. 17.81%, and the JBQG group was significantly lower than the MTX group. The OR value for High between the two groups was 11.56 (95% CI: 3.02–44.25), indicating that the use of JBQG has a significant impact on the occurrence of High. From DAS28-ESR, we found that JBQG has a better clinical effect on recurrent and high-recurrence joint inflammation in RA, which often strengthens the confidence of patients to continue treatment. At the same time, the advantage of the JBQG group can be maintained until 36 weeks after medication, and the two groups tend to be consistent at 60 weeks (Figure 2).

In terms of inflammatory markers, we observed a significant reduction in CRP (8.54 ± 5.87 vs. 11.86 ± 7.92, *p* < 0.05, *p* = 0.005) and ESR (15.1 ± 6.11 vs. 21.96 ± 9.19, *p* < 0.0001) in the JBQG group compared to the MTX group, indicating that the combination of JBQG can significantly control the inflammation level of RA patients. In addition, TNF-α was significantly reduced in the JBQG group compared to the MTX group (1.44 ± 0.83 vs. 1.85 ± 1.07, *p* < 0.05, *p* = 0.011), IL-17 was significantly reduced (0.53 ± 0.33 vs. 0.71 ± 0.38, *p* < 0.05, *p* = 0.004), and INF-γ was significantly reduced (3.2 ± 1.51 vs. 3.89 ± 1.77, *p* < 0.05, *p* = 0.014). By examining the changes in cytokine levels, we can determine the level of inflammation and disease progression in RA patients. After the patient receives treatment, if the cytokine levels in the blood decrease significantly, it may indicate good treatment efficacy; if the cytokine levels remain high, it may indicate poor treatment efficacy and the need to adjust the treatment plan. Although the JBQG group did not decrease IL-1β and IL-6, it did reduce the common inflammatory targets in RA, such as TNF-α, which is still helpful for the clinical treatment of RA.

In addition, we observed changes in OPG and *β*-CTX in the JBQG group. The median (IQR) of OPG was 2.54 (2.21–3.01), which was significantly higher than that in the MTX group 2.06 (1.81–2.32) (*p* < 0.0001); the median (IQR) of *β*-CTX was 0.4 (0.32–0.43), which was significantly lower than that in the MTX group 0.55 (0.47–0.67) (*p* < 0.0001), indicating that the combination of JBQG and MTX can reduce bone turnover rate and inhibit the activation of bone resorption cells from some mechanism. In terms of functional evaluation of RA patients, the median (IQR) of VAS scores was 2 (1–3), which was lower than that in the MTX group 3 (2–4), indicating that Chinese medicine intervention can improve the pain status of patients (*p* < 0.0001).

The median (IQR) of Sharp scores was 1 (1–2), that was lower than that in the MTX group 2 (1–2), but the difference was not statistically significant (*p* > 0.05, *p* = 0.28). Due to the limited observation time, no statistically significant difference was observed in the bone destruction scores of RA. The median (IQR) of HAQ-DI scores was 11 (8–16), which was significantly lower than that in the MTX group 26 (16–30) (*p* < 0.0001), indicating that Chinese medicine intervention has a good effect on improving the quality of life of RA patients. In terms of safety indicators, 9.6% (n = 7) of patients in the MTX group experienced adverse events, the most common reason for withdrawal from the study was drug-related side effects, such as leukopenia and abnormal liver and kidney function indices, so we stopped some patients from continuing the study. In the JBQG group, 2.8% (n = 2) of patients experienced adverse events, all of which were gastrointestinal reactions, such as vomiting and nausea, caused by the patients. Generally, some patients taking traditional Chinese medicine or proprietary Chinese medicine will experience gastrointestinal reactions, which have been reported in other diseases, such as adjuvant treatment of cancer (Cao et al., 2020; Zhang X et al., 2021). In the short term, we observed differences in liver function indices AST and ALT between the two groups, but both remained within the normal range of 20 U/L. We also have evidence that liver function indices in the JBQG group are safer than those in the MTX group. At the same time, no serious adverse events such as death caused by traditional Chinese medicine were observed.

This study was conducted in a relatively small number of RA patients with a relatively short duration, especially in terms of observation of bone destruction. Most patients, under routine treatment, have a significantly prolonged progression of bone destruction, thus, we did not observe such long-term events that require prolonged exposure. Despite these limitations, we still found good clinical efficacy in the JBQG group after treatment and observed that it can inhibit the expression of some cytokines, although we previously observed this in the CIA mouse model, and these findings were also confirmed in this study (Xie et al., 2020). In the objective imaging assessment, we included the Sharp score, but there still remains significant subjectivity. At the same time, because traditional Chinese medicine has complex ingredients, it is difficult to analyze its role from a specific target, and we mainly focus on clinical efficacy and safety for the benefit of RA patients. Therefore, we still need more and longer clinical trials with more RA patients to confirm these findings.

## Data Availability

The raw data supporting the conclusion of this article will be made available by the authors, without undue reservation.
